# Supplementary material for: Training an Ising machine with equilibrium propagation
Source: Nat Commun. 2024 Apr 30;15:3671. doi: 10.1038/s41467-024-46879-4 (PMC11063034; doi:10.1038/s41467-024-46879-4)
Supplement: Supplementary file 1 — Supplementary Information [file 41467_2024_46879_MOESM1_ESM.pdf]

# Supplementary Materials for Training an Ising Machine with Equilibrium Propagation

Jérémie Laydevant[1*], Danijela Marković[1] and Julie Grollier[1*]

[1]Laboratoire Albert Fert, CNRS, Thales, Université Paris-Saclay, 91767 Palaiseau, France.

*Corresponding author(s). E-mail(s): jeremie.laydevant@gmail.com; julie.grollier@cnrs-thales.fr;
Contributing authors: danijela.markovic@cnrs-thales.fr;

**Abstract**

We present here the supplementary materials for Training an Ising Machine with Equilibrium Propagation.

## Contents

**1 Equilibrium Propagation training loop** **3**

**2 State-of-the-art performance of Equilibrium Propagation** **4**

**3 Performance of different "hardware-compatible" learning algorithms - including Equilibrium Propagation** **5**

**4 Qualitative and quantitative comparison of different hardware implementations of Ising Machines** **5**

**5 Simulated annealing** **7**

**6 Deterministic dynamics** **7**

**7 Nudging phase with an Ising machine** **7**

**8 Fully connected architecture - D-Wave - SA - Deterministic** **10**

8.1 Embedding of the fully-connected architecture on the chip . . . 10
8.2 Training curves - D-Wave . . . . . . . . . . . . . . . . . . . . . 10
8.3 Training curves - SA . . . . . . . . . . . . . . . . . . . . . . . 11
8.4 Parameters: . . . . . . . . . . . . . . . . . . . . . . . . . . . . 12

**9 Convolutional architecture - D-Wave - SA - Deterministic 13**
9.1 Training curves - D-Wave . . . . . . . . . . . . . . . . . . . . . 14
9.2 Training curves - Simulated Annealing . . . . . . . . . . . . . . 14
9.3 Embedding . . . . . . . . . . . . . . . . . . . . . . . . . . . . . 16
9.4 Parameters . . . . . . . . . . . . . . . . . . . . . . . . . . . . . 17

# 1 Equilibrium Propagation training loop

**Supplementary Algorithm 1** Generic Equilibrium Propagation training loop - the energy minimization depends on the hardware and/ or algorithm used.

**Input**: Input data $x$ and the corresponding target $\hat{y}$, initial state of the system $s$, nudging parameter $\beta$, Set of parameters $\theta = \{W, b\}$, Energy function of the system (Ising, Hopfield, etc.), learning rate $\eta$

**Output**: Updated set of parameters $\theta = \{W, b\}$

***1. Free phase***

$s^{*,0} = \arg\min_s E(\theta, s, x)$ ▷ Get the minimum of E given $x$

***2. Free phase***

$s^{*,\beta} = \arg\min_s E(\theta, s, x, \beta, \hat{y}) + \beta \cdot C(y, \hat{y})$ ▷ Get the minimum of E given $x, \beta, \hat{y}$

***3. Compute the gradient***

$\frac{\partial \mathcal{L}}{\partial \theta} = \frac{1}{\beta} \left( \frac{\partial E}{\partial \theta}(x, \theta, \rho(s)^{*,\beta}, \beta, \hat{y}) - \frac{\partial E}{\partial \theta}(x, \theta, \rho(s)^{*,0}, 0, 0) \right)$

***4. Update the parameters with SGD***

$\theta \leftarrow \theta - \eta \frac{\partial \mathcal{L}}{\partial \theta}$

# 2 State-of-the-art performance of Equilibrium Propagation

We report here the different accuracies reached with Equilibrium Propagation and backpropagation through time found in the literature for image classification and natural language processing tasks.

**Supplementary Table 1** Test accuracy on different image classification benchmark tasks found in the literature

| | MNIST | | | | CIFAR-10 [1] | | CIFAR-100 [2] | Image-nette 32x32 [1] | |
|---|---|---|---|---|---|---|---|---|---|
| | MLP [3] | | Conv [2] | | Conv | | Conv | Conv | |
| | EP | BPTT | EP | BPTT | EP | BPTT | EP | EP | BPTT |
| Test accuracy (%) | 98 | 98 | 99.58 | 99.58 | 90.3 | 89.9 | 68.4 | 36.5 | 37.2 |

**Supplementary Table 2** Test accuracy on different natural language processing benchmark tasks found in the literature

| | IMDB [4] | | SNLI [4] | |
|---|---|---|---|---|
| | Modified Modern Hopfield Network | | | |
| | EP | BPTT | EP | BPTT |
| Test accuracy (%) | 88.9 | 88.4 | 81.4 | 80.9 |

# 3 Performance of different "hardware-compatible" learning algorithms - including Equilibrium Propagation

| | PEPITA [5] | FA [6] | DFA [6] | Softhebb [7] | CL [8] | |
|---|---|---|---|---|---|---|
| Architecture | 1 conv layer | 3 conv layers | 3 conv layers | 3 conv layers | 4 conv layers | |
| Test error (%) | 43.67 | 27.1 | 26.9 | 19.7 | 13.5 | |
| | EP [2] | RBP[2] | BPTT [2] | TP [9] | SP [10] | CHLD [11] |
| Architecture | 4 conv layers | 4 conv layers | 4 conv layers | 5 conv layers | VGG8 | VGG16 |
| Test error (%) | 11.1 | 10.7 | 10.1 | 10.4 | 8.34 | 7.7 |

**Supplementary Table 3** Performance achieved on CIFAR-10 by some hardware-compatible learning algorithms

Abbreviations used:

- FA: Feedback Aligment
- DFA: Direct Feedback Aligment
- CL: Coupled Learning
- EP: Equilibrium Propagation
- RBP: Recurrent BackPropagation
- BPTT: BackPropagation Through Time
- SP: Signal Propagation
- CHLD: Contrastive Hebbainn Learning with Diadic neurons

# 4 Qualitative and quantitative comparison of different hardware implementations of Ising Machines

| **Technology** | **Physical Encoding of the Ising Energy** | **Implementation of the spin dynamics** | **Connectivity (implementation)** | **Previous usage Combinatorial optimization/ ML/ Other** | ML algorithm used | **Availability/ interface** |
|---|---|---|---|---|---|---|
| Coherent-IM [12] | YES: Optical Loss | Optical pulses encoding spins in the phase - non-linear dynamics with phase-dependent gain/ Time-multiplexed | All-to-all (up to 100 000 spins [13]) (Measurement-feedback couplings with a side FPGA) | Combinatorial optimization | Non reported | Limited: experimental work/ unknown |
| Poor's man-IM [14] | NO | Mixed digital-optical implementation: discrete Euler updates realized through a non-linear electro-optical modulator/ Time-multiplexed | All-to-all (1 000 spins) (Measurement-feedback couplings with a side FPGA) | ML: Pre-trained a Boltzmann machine/ MCMC acceleration | Contrastive divergence + backpropagation [15] (sub-sampled MNIST) | Limited: experimental work/ unknown |
| Memristive-IM [16] | NO | Digital spins updated with hysteretic thresholding the current flowing from the crossbar array/ Parallel updates | All-to-all (60 spins) (Direct coupling through a crossbar array) | Combinatorial optimization/ Training a Hopfield network to reconstruct and classifying braille sentences [17] | MADEM [17] (close to EqProp) | Limited: experimental work/ unknown |
| FPGA-IM (Fujitsu) [18] | NO | Digital spins updated according to Glauber dynamics/ Individual updates | All-to-all (8 192 spins) (Measurement-feedback couplings) | Combinatorial optimization ML: unknown use | Non reported | Yes: commercially available as a remote ressource (cloud)/ Poor API no python interfacability |
| FPGA-IM (Toshiba) [19] | YES: Simulated loss of coupled oscillators | Digital spins with simulated bifurcation dynamics | All-to-all (8-coupled FPGAs: 8 192 spins [19]) GPU: under development) (Measurement-feedback couplings) | Combinatorial optimization/ ML: unknown use | Non reported | Yes: commercially available as a remote ressource (cloud)/ Poor API no python interfacability |
| CMOS-IM [20] | YES: Phase interaction (Kuramoto model) | Spins encoded in the phase of the electrical oscillators/ Parallel dynamics | All-to-all (48 spins) (Direct coupling) | Combinatorial optimization | Non reported | Limited: experimental work/ unknown |
| Superconducting-IM [21] | YES: Hamiltonian of the system | Spins encoded in the circulation of a superconducting current/ Parallel dynamics | Local: Chimera (2042 spins) or Pegasus (5640 spins) (Couplings through local SQUIDs) | Combinatorial optimization/ ML: [22] pre-trained a Boltzmann machine | Contrastive Divergence + backpropagation [22] (sub-sampled MNIST) | Yes: commercially available as a remote ressource (cloud)/ Python API with calls to the solver very simple interface |

**Supplementary Table 4** Different hardware implementations to solve the Ising problem

# 5 Simulated annealing

Quite remarkably, we have been able to use almost the same parameters that we have used for the D'Wave trainings.

In order to benchmark the trainings conducted on the D-Wave Ising machine, we performed two types of digital simulations.

The first type of digital simulation we performed was based on Simulated Annealing, which is conceptually similar to quantum annealing. In this case, the system of coupled spins is driven towards the ground state of the Ising energy function through controlled, decreasing thermal effects. We based our simulations on the Simulated Annealing code provided by D-Wave (https://github.com/jlaydevant/Ising-Machine-EqProp) because of its native speed. However, just as the D-Wave code extensively uses the auto-scale feature to optimize the accessible range of parameters, the Simulated Annealing code also employs auto-scaling by default. This auto-scaling adjusts the initial and final temperature of the annealing schedule according to the parameters of the Ising energy function.

As previously discussed, we wanted to avoid the auto-scale feature because it can result in the nudge phase not being exactly equivalent to the free phase plus the cost function to minimize. Therefore, we adapted the initial code to one that maintains the same temperature schedule for all free and nudge phases. We also had to define the equivalent of the annealing fraction used for determining how "far" we go during the nudge phase. Remarkably, we were able to use almost the same parameters for these simulations as those utilized for the D-Wave trainings.

The SA algorithm works in the following way:

# 6 Deterministic dynamics

The second kind of digital simulations we have performed are based on the initial EP dynamics where the energy function is similar to that of standard EP, but the activations are binary (0/1) and the synaptic weights are real-valued. These simulations are derived from the work about training BNNs with EP [23]. The dynamics is then purely deterministic regarding the initial state.

# 7 Nudging phase with an Ising machine

Here we show how we can derive the nudging term when the energy function that the system minimizes is the Ising energy function.

The energy function, based on the Ising energy function (Eq. (**??**)), for the nudge phase is the following:

$$E_{Ising} + \beta \cdot C(\sigma(y), \sigma(\hat{y})) = \sum_{i>j} J_{ij}\sigma_i\sigma j + \sum_i h_i\sigma_i + \frac{\beta}{2}\sum_{i\in Y}(\sigma(y_i) - \sigma(\hat{y}_i))^2 \quad (1)$$

**Supplementary Algorithm 2** Simulated annealing

**Input**: Graph $G$ with spins $\{\sigma_i\}_{i\in G}$, local couplings $J_{ij}$, Temperature annealing schedule (initial and final $T$ + cooling law): $\{T^0,T^f\}$
**Output**: $\sigma_{ground} = \{\sigma_i\}_{i\in G}$
**for** $T \in [T^0, T^f]$ **do**
  **for** $t \in [0, t_{per\ T}]$ **do**
    1. Randomly choose a spin $\sigma_0^{x,y}$ whose coordinates are $x, y$
    2. Compute $\Delta E$ if $\sigma^{x,y}$ flips:
      $\Delta E = (\sigma_{flip}^{x,y} - \sigma_0^{x,y}) * [J_{x,y;\ x+1,y}\sigma^{x+1,y} + J_{x,y;\ x-1,y}\sigma^{x-1,y}$
      $+J_{x,y;\ x,y-1}\sigma^{x,y-1} + J_{x,y;\ x,y+1}\sigma^{x,y+1}]$
      and since $\sigma_{flip}^{x,y} = -\sigma_0^{x,y}$ we can simplify:
      $\Delta E = -2\sigma_0^{x,y} * [J_{x,y;\ x+1,y}\sigma^{x+1,y} + J_{x,y;\ x-1,y}\sigma^{x-1,y}$
      $+J_{x,y;\ x,y-1}\sigma^{x,y-1} + J_{x,y;\ x,y+1}\sigma^{x,y+1}]$
    3. Decision to flip the spin:
    **if** $\Delta E < 0$ **then** ▷ Gradient Descent on the energy
      $\sigma^{x,y} \leftarrow -\sigma^{x,y}$
    **else**
      $\sigma^{x,y} \leftarrow -\sigma^{x,y}$ with $p \propto e^{-\frac{\Delta E}{T}}$ ▷ Boltzmann probability
    **end if**
  **end for**
**end for**

**Supplementary Algorithm 3** Deterministic dynamics for EP training

**Input**: Input data $x$ and the corresponding target $\hat{y}$, initial state of the system $s$, nudging parameter $\beta$
**Output**: $s^*$
**for** $t \in [0, T]$ **do**
  **if** $\beta = 0$ **then**
    $s \leftarrow s - dt \cdot \frac{\partial E}{\partial s}(\theta, s, x)$
  **else**
    $s \leftarrow s - dt \cdot \left[\frac{\partial E}{\partial s}(\theta, s, x) + \beta \cdot \frac{\partial C}{\partial s}(y, \hat{y})\right]$
  **end if**
**end for**

where, as already described in the main text, the cost function is defined on the binary states of the spins and not their "pre-activation" as conventionally done with EP because we do not have access to this "pre-activation" in the case of the D-Wave Ising machine. Here the sum over $Y$ refers to the output neurons as only their states affect the cost function.

Here we show that we can translate the cost function to be minimized by the system to a simple nudging term that can be easily applied to the Ising machine.

We can expand the last quadratic term of Eq. (1):

$$E_{Ising}+\beta\cdot C(\sigma(y),\sigma(\hat{y})) = \sum_{i>j} J_{ij}\sigma_i\sigma j+\sum_i h_i\sigma_i+\frac{\beta}{2}\sum_{i\in Y}(\sigma(y_i)^2+\sigma(\hat{y}_i)^2-2\sigma(y_i)\sigma(\hat{y}_i)) \tag{2}$$

We can simplify Eq. (2) because the spins always take the values $\pm 1$, so $\sigma(y_i)^2 = 1$ and $\sigma(\hat{y}_i)^2 = 1$:

$$\begin{aligned} E_{Ising} + \beta \cdot C(\sigma(y),\sigma(\hat{y})) &= \sum_{i>j} J_{ij}\sigma_i\sigma j + \sum_i h_i\sigma_i + \frac{\beta}{2}\sum_{i\in Y}(2 - 2\sigma(y_i)\sigma(\hat{y}_i)) \\ &= \sum_{i>j} J_{ij}\sigma_i\sigma j + \sum_i h_i\sigma_i + \beta\cdot\sum_{i\in Y}(1 - \sigma(y_i)\sigma(\hat{y}_i)) \end{aligned} \tag{3}$$

We can remove the last term which is a simple offset term, and because the ground state does not depend on that term, and interpret the term $\beta \cdot \sigma(\hat{y}_i)$ as a nudging bias applied to spin $\sigma(y_i)$:

$$E_{Ising} + \beta \cdot C(\sigma(y),\sigma(\hat{y})) = \sum_{i>j} J_{ij}\sigma_i\sigma j + \sum_{i\notin Y} h_i\sigma_i + \sum_{i\in Y}(h_i - \beta\cdot\sigma(\hat{y}_i))\sigma_i \tag{4}$$

Here the sum over $Y$ refers to the output neurons to which we apply nudging biases and the sum over $\notin Y$ refers to the other neurons in the network - i.e., the hidden neurons. This second energy function, close to the one of the free phase, is minimized by the Ising machine through the reverse annealing procedure in order to reach the second equilibrium state. Thanks to the binary nature of the spins, the nudge is simply carried out by applying the nudging biases to the output neurons.

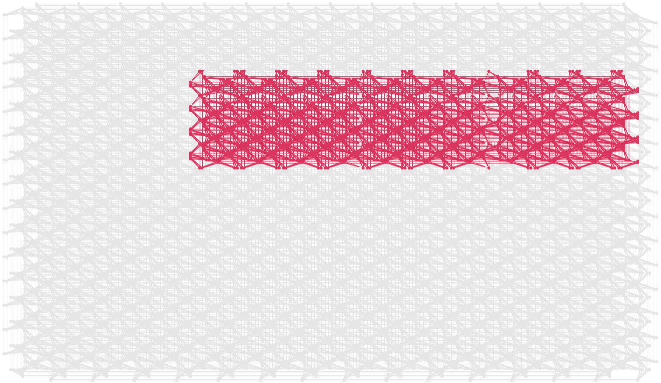

**Supplementary Fig. 1** Embedding of the fully-connected architecture on the Advantage chip. The pink nodes stand for the spins on the chip and the edges refer to the couplings between the spins.

# 8 Fully connected architecture - D-Wave - SA - Deterministic

## 8.1 Embedding of the fully-connected architecture on the chip

## 8.2 Training curves - D-Wave

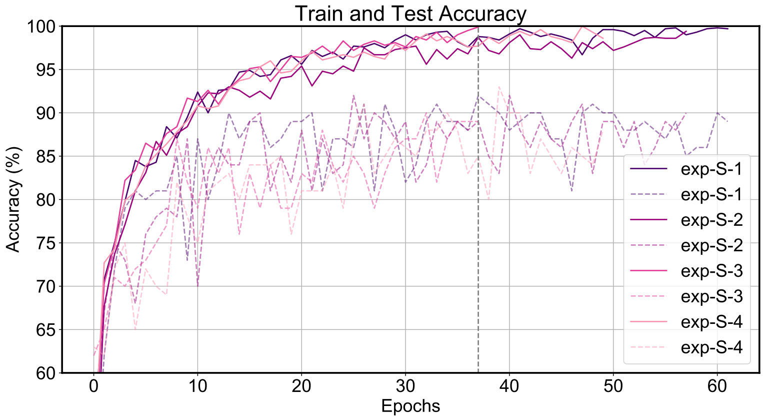


**Supplementary Fig. 2** Train (solid) and test (dashed) accuracy vs epochs for different trainings. We indicate with the dashed gray line the epoch to which we report the average accuracy in the main text.

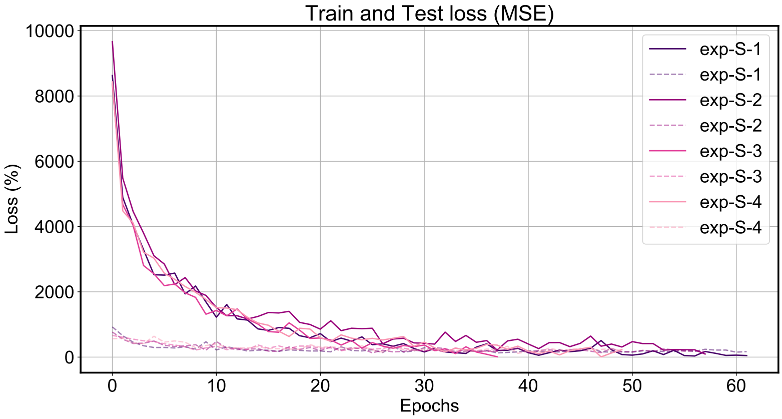


**Supplementary Fig. 3** Train (solid ) and test (dashed) loss (MSE) vs epochs for different trainings.

## 8.3 Training curves - SA

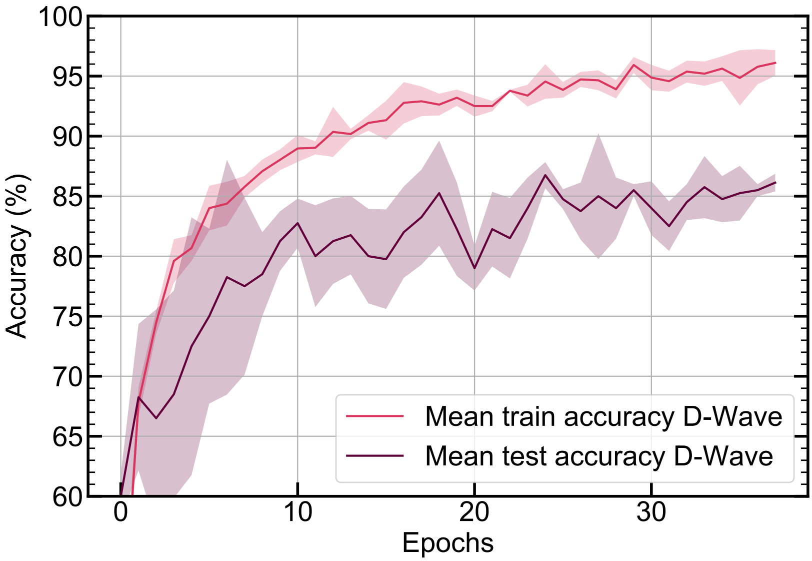


**Supplementary Fig. 4** Train (solid) and test (dashed) mean accuracy vs epochs. We report the accuracy reached after the same number of epochs as used on the D-Wave Ising machine.

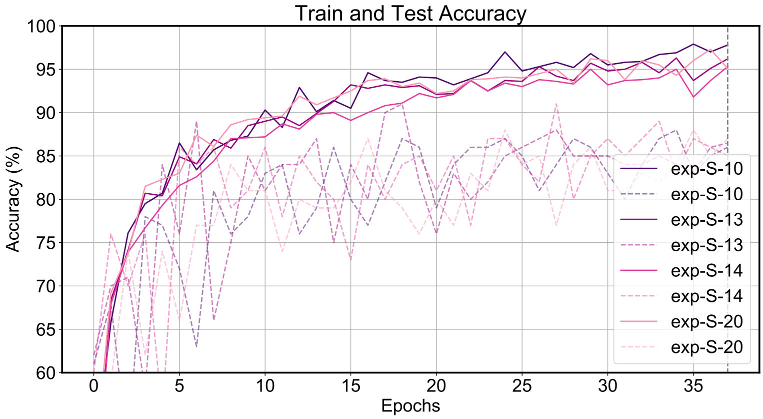


**Supplementary Fig. 5** Train (solid) and test (dashed) accuracy vs epochs for different trainings.

## 8.4 Parameters:

**Supplementary Table 5** Hyperparameters used for the trainings on D-Wave (same parameters used for SA)

| Parameter | $N_{\text{free}}$ | $N_{\text{nudge}}$ | Beta | $lr_{\text{W}}$ | $lr_{\text{B}}$ | Scaling weight input | Scaling weight chip |
|---|---|---|---|---|---|---|---|
| Value | 10 | 10 | 2 | 1e-2 | 1e-3 | 0.5 | 0.25 |

**Supplementary Table 6** Parameters used for the trainings on D-Wave - chip's parameters (same parameters used for SA)

| Parameter | Chain strengh | Range J | Range h | Forward annealing time | Reverse annealing time | Fraction reverse annealing |
|---|---|---|---|---|---|---|
| Value | 1 | [-2,+2] | [-4,+4] | 20 $\mu s$ | 40 $\mu s$ | 0.25 |

**Supplementary Table 7** Hyperparameters used for the trainings with the deterministic dynamics

| Parameter | $T$ | $K$ | dt | Beta | $lr_{\text{W}}$ | $lr_{\text{B}}$ |
|---|---|---|---|---|---|---|
| Value | 30 | 50 | 0.5 | 2 | 1e-1 | 1e-2 |

# 9 Convolutional architecture - D-Wave - SA - Deterministic

Contrarily to the trainings of the fully-connected architecture on the Ising machine where the embedding was done through the LazyFixedEmbedding procedure provided by D-Wave, we here designed a custom embedding so that the convolutional architecture is really on the Ising machine.

To that end, we need to specify the correspondence of a specific neuron from the convolutional architecture to a specific spin on the layout. We have chosen to embed the architecture in a region where no faulty spins have been reported (we can know which spin is faulty through the list of the indices of the nodes available on the chip we are using).

Now we need to distinguish the different kind of neurons we need to embed:

- The input nodes: they are individual spins that are strongly biased (h=+/-2 - the maximal value on the 2000Q chip) in order to represent the input pixel's binary value. As the different windows on the input image overlap, we need to set the same bias value to different spins on the chip in order to represent the same pixel value.
- The convolution neurons: they are the spins coupled to the input nodes and represent the output of the local multiplication of the convolutional filter and the patch of the input data. They require a single spin per neuron.
- The averaged pooling neurons: those neurons need to collect information from all the neurons (spins) that result from the convolutional operation. As they are spatially distant, we need to chain multiple spins on the chip to create a "bus" that is coupled through average couplings $\frac{1}{4}$ to the convolution neurons and carries the information of the Averaged pooling operation.
- The output neurons: finally, the state of the Average pooling chains are fed to a simple fully connected classifier where 4 output neurons encode the output of the network - and more precisely, 2 output neuron/ class are used to predict the class of the input and for the nudging phase.

This results in the following embedding dictionary: *embedding* where the indices of the neurons can be found in Fig. S11.

## 9.1 Training curves - D-Wave

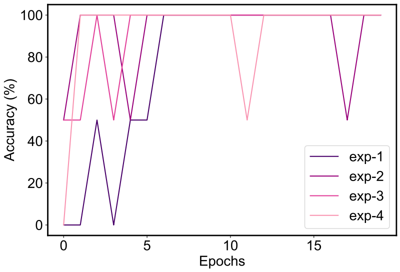


**Supplementary Fig. 6** Train accuracy vs epochs for different trainings on the D-Wave Ising machine.

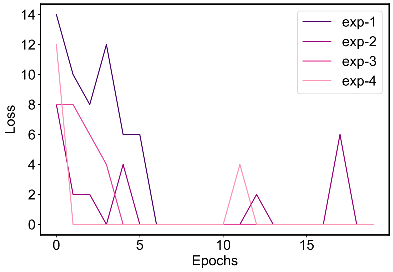


**Supplementary Fig. 7** Train loss (MSE) vs epochs for different trainings on the D-Wave Ising machine.

## 9.2 Training curves - Simulated Annealing

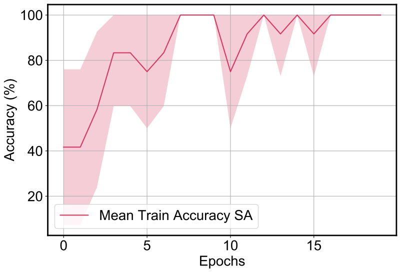


**Supplementary Fig. 8** Mean train accuracy vs epochs for trainings with SA.

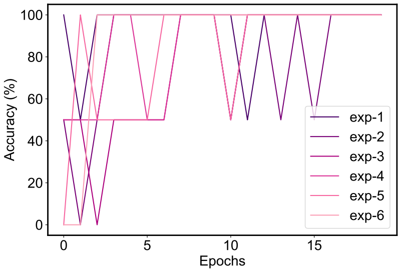


**Supplementary Fig. 9** Train accuracy vs epochs for different trainings with SA.

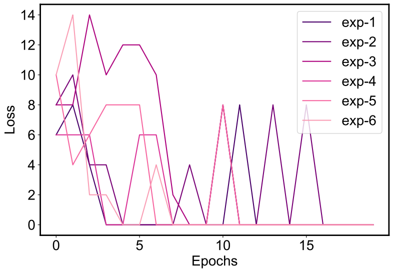


**Supplementary Fig. 10** Train loss (MSE) vs epochs for different trainings with SA.

## 9.3 Embedding

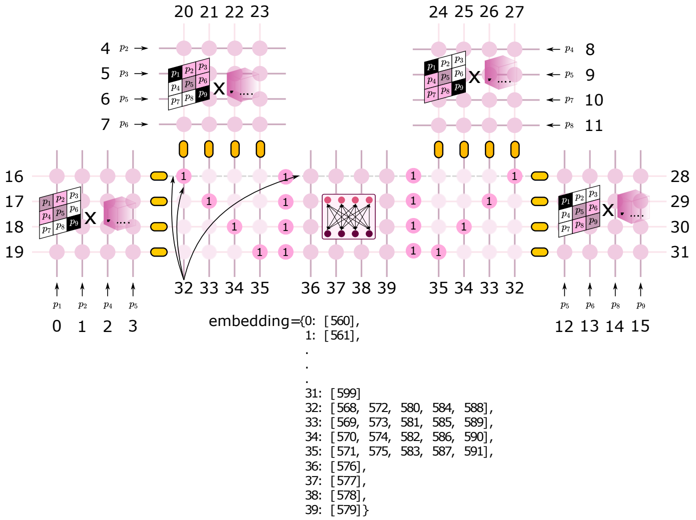


**Supplementary Fig. 11** Neuron to qubit index on the D-Wave chip. Chained spins required a special treatment as we use mulitple hardware spins to represent a single neuron. The embedding dictionary we show has its keys being the index a the neuron and the corresponding entry the index(ices) of the hardware spins.

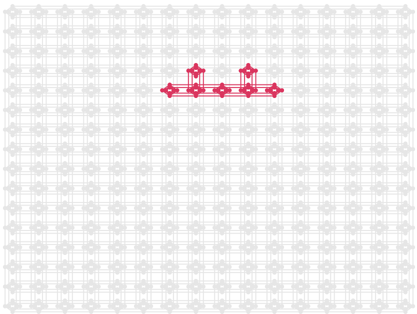

**Supplementary Fig. 12** Embedding of the convolutional neural network on the DW-2000 D-Wave Ising machine. The pink nodes stand for the spins on the chip and the edges refer to the couplings between the spins. This embedding is the embedding depicted in Fig. 11.

## 9.4 Parameters

**Supplementary Table 8** Parameters describing the convolutional architecture we trained on the D-Wave Ising machine

| Parameter | Kernel size | Stride | Padding | Pooling size | Avg Pool coef | Classifier |
|---|---|---|---|---|---|---|
| Value | 2 | 1 | 0 | 2 | $\frac{1}{4}$ | 4x4 |

**Supplementary Table 9** Hyperparameters used for the trainings of the convolutional architecture on the D-Wave Ising machine

| Parameter | $N_{free}$ | $N_{nudge}$ | Beta | $lr_{W_{conv}}$ | $lr_{W_{class}}$ | Scaling weight conv | Scaling weight class |
|---|---|---|---|---|---|---|---|
| Value | 10 | 10 | 5 | 1e-1 | 1e-1 | 0.1 | 0.1 |

**Supplementary Table 10** Parameters used for the trainings of the convolutional architecture on the D-Wave Ising machine - chip's parameters

| Parameter | Chain strengh | Range J | Range h | Forward annealing time | Reverse annealing time | Fraction reverse annealing |
|---|---|---|---|---|---|---|
| Value | 2 | [-1,+1] | [-4,+4] | 20 $\mu s$ | 40 $\mu s$ | 0.25 |

# Supplementary References

[1] Laborieux, A., Zenke, F.: Holomorphic equilibrium propagation computes exact gradients through finite size oscillations. In: Koyejo, S., Mohamed, S., Agarwal, A., Belgrave, D., Cho, K., Oh, A. (eds.) Advances in Neural Information Processing Systems, vol. 35, pp. 12950–12963. Curran Associates, Inc., (2022). https://proceedings.neurips.cc/paper_files/paper/2022/file/545a114e655f9d25ba0d56ea9a01fc6e-Paper-Conference.pdf

[2] Scellier, B., Ernoult, M., Kendall, J., Kumar, S.: Energy-based learning algorithms for analog computing: a comparative study. In: Thirty-seventh Conference on Neural Information Processing Systems (2023). https://openreview.net/forum?id=jl5a3t78Uh

[3] Ernoult, M., Grollier, J., Querlioz, D., Bengio, Y., Scellier, B.: Updates of equilibrium prop match gradients of backprop through time in

an rnn with static input. In: Wallach, H., Larochelle, H., Beygelzimer, A., d' Alché-Buc, F., Fox, E., Garnett, R. (eds.) Advances in Neural Information Processing Systems, vol. 32. Curran Associates, Inc., (2019). https://proceedings.neurips.cc/paper/2019/file/67974233917cea0e42a49a2fb7eb4cf4-Paper.pdf

[4] Bal, M., Sengupta, A.: Sequence Learning using Equilibrium Propagation (2023). https://doi.org/10.48550/arXiv.2209.09626

[5] Dellaferrera, G., Kreiman, G.: Error-driven input modulation: Solving the credit assignment problem without a backward pass. In: Chaudhuri, K., Jegelka, S., Song, L., Szepesvari, C., Niu, G., Sabato, S. (eds.) Proceedings of the 39th International Conference on Machine Learning. Proceedings of Machine Learning Research, vol. 162, pp. 4937–4955. PMLR, ??? (2022). https://proceedings.mlr.press/v162/dellaferrera22a.html

[6] Nøkland, A.: Direct Feedback Alignment Provides Learning in Deep Neural Networks. arXiv (2016). https://doi.org/10.48550/arXiv.1609.01596. http://arxiv.org/abs/1609.01596

[7] Journé, A., Rodriguez, H.G., Guo, Q., Moraitis, T.: Hebbian deep learning without feedback. In: The Eleventh International Conference on Learning Representations (2023). https://openreview.net/forum?id=8gd4M-_Rj1

[8] Stern, M., Hexner, D., Rocks, J.W., Liu, A.J.: Supervised learning in physical networks: From machine learning to learning machines. Physical Review X **11**(2), 021045 (2021). https://doi.org/10.1103/PhysRevX.11.021045

[9] Ernoult, M.M., Normandin, F., Moudgil, A., Spinney, S., Belilovsky, E., Rish, I., Richards, B., Bengio, Y.: Towards scaling difference target propagation by learning backprop targets. In: International Conference on Machine Learning, pp. 5968–5987 (2022). PMLR. https://proceedings.mlr.press/v162/ernoult22a/ernoult22a.pdf

[10] Kohan, A., Rietman, E.A., Siegelmann, H.T.: Signal propagation: The framework for learning and inference in a forward pass. IEEE Transactions on Neural Networks and Learning Systems, 1–12 (2023). https://doi.org/10.1109/TNNLS.2022.3230914

[11] Høier, R., Staudt, D., Zach, C.: Dual propagation: Accelerating contrastive hebbian learning with dyadic neurons. arXiv preprint arXiv:2302.01228 (2023)

[12] Yamamoto, Y., Aihara, K., Leleu, T., Kawarabayashi, K.-i., Kako, S., Fejer, M., Inoue, K., Takesue, H.: Coherent Ising machines—optical neural networks operating at the quantum limit. npj Quantum Information **3**(1),

49 (2017). https://doi.org/10.1038/s41534-017-0048-9

[13] Honjo, T., Sonobe, T., Inaba, K., Inagaki, T., Ikuta, T., Yamada, Y., Kazama, T., Enbutsu, K., Umeki, T., Kasahara, R., Kawarabayashi, K.-i., Takesue, H.: 100, 000-spin coherent ising machine. Science Advances **7**(40) (2021). https://doi.org/10.1126/sciadv.abh0952

[14] Böhm, F., Verschaffelt, G., Van der Sande, G.: A poor man's coherent ising machine based on opto-electronic feedback systems for solving optimization problems. Nature Communications **10**(1) (2019). https://doi.org/10.1038/s41467-019-11484-3

[15] Böhm, F., Alonso-Urquijo, D., Verschaffelt, G., der Sande, G.V.: Noise-injected analog ising machines enable ultrafast statistical sampling and machine learning. Nature Communications **13**(1) (2022). https://doi.org/10.1038/s41467-022-33441-3

[16] Cai, F., Kumar, S., Van Vaerenbergh, T., Sheng, X., Liu, R., Li, C., Liu, Z., Foltin, M., Yu, S., Xia, Q., Yang, J.J., Beausoleil, R., Lu, W.D., Strachan, J.P.: Power-efficient combinatorial optimization using intrinsic noise in memristor Hopfield neural networks. Nature Electronics **3**(7) (2020). https://doi.org/10.1038/s41928-020-0436-6

[17] Yi, S.-i., Kendall, J.D., Williams, R.S., Kumar, S.: Activity-difference training of deep neural networks using memristor crossbars. Nature Electronics (2022). https://doi.org/10.1038/s41928-022-00869-w

[18] Tsukamoto, S., Takatsu, M., Matsubara, S., Tamura, H.: An accelerator architecture for combinatorial optimization problems (2017). https://www.fujitsu.com/global/documents/about/resources/publications/fstj/archives/vol53-5/paper02.pdf

[19] Tatsumura, K., Dixon, A.R., Goto, H.: Fpga-based simulated bifurcation machine. In: 2019 29th International Conference on Field Programmable Logic and Applications (FPL), pp. 59–66 (2019). https://doi.org/10.1109/FPL.2019.00019

[20] Lo, H., Moy, W., Yu, H., Sapatnekar, S., Kim, C.H.: An ising solver chip based on coupled ring oscillators with a 48-node all-to-all connected array architecture. Nature Electronics (2023). https://doi.org/10.1038/s41928-023-01021-y

[21] Harris, R., Johnson, M.W., Lanting, T., Berkley, A.J., Johansson, J., Bunyk, P., Tolkacheva, E., Ladizinsky, E., Ladizinsky, N., Oh, T., Cioata, F., Perminov, I., Spear, P., Enderud, C., Rich, C., Uchaikin, S., Thom, M.C., Chapple, E.M., Wang, J., Wilson, B., Amin, M.H.S., Dickson, N., Karimi, K., Macready, B., Truncik, C.J.S., Rose, G.: Experimental

investigation of an eight-qubit unit cell in a superconducting optimization processor. Phys. Rev. B **82**, 024511 (2010). https://doi.org/10.1103/PhysRevB.82.024511

[22] Adachi, S.H., Henderson, M.P.: Application of quantum annealing to training of deep neural networks. arXiv preprint arXiv:1510.06356 (2015). https://doi.org/10.48550/arXiv.1510.06356

[23] Laydevant, J., Ernoult, M., Querlioz, D., Grollier, J.: Training dynamical binary neural networks with equilibrium propagation. In: Proceedings of the IEEE/CVF Conference on Computer Vision and Pattern Recognition (CVPR) Workshops, pp. 4640–4649 (2021). https://openaccess.thecvf.com/content/CVPR2021W/BiVision/papers/Laydevant_Training_Dynamical_Binary_Neural_Networks_With_Equilibrium_Propagation_CVPRW_2021_paper.pdf
